# Enhanced detection of chronic wasting disease in muscle tissue harvested from infected white-tailed deer employing combined prion amplification assays

**DOI:** 10.1371/journal.pone.0309918

**Published:** 2024-10-23

**Authors:** Caitlyn N. Kraft, David W. Bissinger, Erin E. McNulty, Nathaniel D. Denkers, Candace K. Mathiason

**Affiliations:** Department of Microbiology, Immunology and Pathology, Prion Research Center, College of Veterinary Medicine and Biomedical Sciences, Colorado State University, Fort Collins, Colorado, United States of America; National Institute of Allergy and Infectious Diseases, UNITED STATES OF AMERICA

## Abstract

Zoonotic transmission of bovine spongiform encephalopathy or mad cow disease, by presumed consumption of infected beef, has increased awareness of the public health risk associated with prion diseases. Chronic wasting disease (CWD) affects moose, elk, and deer, all of which are frequently consumed by humans. Clear evidence of CWD transmission to humans has not been demonstrated, yet, establishing whether CWD prions are present in muscle tissue preferentially consumed by humans is of increasing interest. Conventional assays including immunohistochemistry (IHC) and enzyme-linked immunosorbent assay (ELISA) lack the sensitivity to detect low concentrations of prions presumed to be present outside neural or lymphatic tissues. Here we combined two prion amplification assays, the product of protein misfolding cyclic amplification (PMCA) applied directly into real-time quaking induced conversion (RT-QuIC) [denoted now as PQ] to demonstrate the presence of prion seeding activity (i.e. prions) in ~55% of hamstring muscles harvested from CWD-positive white-tailed deer. This compares to prion detection in only 10% of the same samples employing standard RT-QuIC. To determine the extent of CWD dissemination within muscle tissues commonly consumed we tested 7 additional muscles from a subset of deer by PQ. Tongue demonstrated the highest level of prions with ~92% positive. All negative controls remained negative in all PMCA and RT-QuIC assays. We conclude that the combination of PMCA with RT-QuIC readout permits detection of low prion concentrations present in muscle tissue of CWD-infected deer. These findings further demonstrate the utility of amplification assays as tools to detect very low levels of prion burden and supports their use to fill knowledge gaps in our understanding of CWD pathogenesis and zoonotic potential.

## Introduction

Transmissible spongiform encephalopathies, or prion diseases, affect a variety of mammalian species, including humans. Creutzfeldt Jakob disease (CJD) in humans is known to be acquired via genetic or iatrogenic means [1, 2]. Variant CJD (vCJD), identified shortly after the Bovine Spongiform Encephalopathy (BSE) outbreak in the 1980s, has been associated with the consumption of meat from BSE-infected cattle [3]. This has raised concern for the development of additional zoonotic prion transmissions to humans through the consumption of contaminated meat from other prion-infected species. Cervids, including several species of deer, elk, and moose, are relied upon as a protein source in many communities. Chronic wasting disease (CWD), the prion disease of captive and free-ranging cervid populations in North America, Asia, and Europe, continues to spread [4]. It has been demonstrated that prion deposition is widely distributed within tissues and bodily fluids of CWD-infected animals throughout the long asymptomatic phase of disease (a phase that can last 2–3 years, or an entire lifespan) [5–7]. Thus, CWD prions are most assuredly being ingested by those that consume venison.

In the case of BSE, cows presumed infected through repeated oral exposure to meat and bone meal sourced from cattle with spontaneous BSE infections [8]. As muscle is the primary bovine tissue consumed by humans, detection of BSE positivity in muscle tissue was of prime importance. Extensive research has gone into detection of BSE in muscle tissues of cattle, as well as analyzing the infectivity of these samples. In bioassay, BSE-susceptible mice were infected with muscle tissue from infected cattle, including muscle tissues that were negative by immunoblot. These mice revealed signs of clinical prion disease, suggesting the presence of the infectious agent within muscle tissue [9]. Prion deposition was detected in triceps muscle spindles of H-BSE infected cattle, medial gluteal muscle of L-BSE infected cattle, and throughout the body of cattle infected with classical BSE [10, 11] when assessed by immunohistochemistry (IHC). Overall, BSE deposition in muscle tissues is at significantly lower concentrations than that demonstrated within the central nervous system and was only detected in cattle with clinical signs of disease.

Considerable effort has been dedicated to characterizing the presence and distribution of CWD deposition in cervid tissues, including muscle. Conventional assays, including IHC, enzyme-linked immunosorbent assay (ELISA), and western blotting (WB), permitted detection of CWD prion protein (PrP^CWD^) deposition in cardiac myocytes of the left ventricle harvested from white-tailed deer and elk [12], and within skeletal muscle tissues of elk as they approached terminal clinical disease [13]. Further evidence for the presence of infectious prions in muscle tissue was revealed by mouse bioassay of CWD-infected mule deer cardiac myocytes [14]. Incorporation of the amplification assay, protein misfolding cyclic amplification with western blot readout (PMCA), led to the detection of CWD in skeletal muscle, though at a 2000 to 10000 fold lower concentration as compared with brain tissue [15]. Further modification of prion amplification assays, including sample pre-treatment with a combination of sodium phosphotungstic acid (NaPTA) precipitation and freeze-thaw cycles, were used in conjunction with real-time quaking induced conversion (RT-QuIC) to test commonly consumed muscle tissues from free-ranging white-tailed deer. This led to the detection of prion seeding activity in 8 of 10 neck tissues, and 14.7% of other tissues throughout the periphery [16]. Yet, little is known about the presence of CWD prions in cervid muscle tissues commonly consumed by humans.

Here, we characterize prion burden in cervid muscle tissue commonly consumed by humans by first employing PMCA amplification, then applying that product directly as the seed into RT-QuIC from CWD-infected white-tailed deer of known CWD status.

## Methods

### Ethics statement

All animals were handled in strict accordance with guidelines for animal care and use provided by the United States Department of Agriculture (USDA), NIH and the Association for Assessment and Accreditation of Laboratory Animal Care International (AAALAC, and all animal work was approved by Colorado State University Institutional Animal Care and Use Committee (IACUC protocol numbers 1466, 10–2189, 12-3773A, 13-4610A).

### White-tailed deer

White-tailed deer (WTD) that were part of previous chronic wasting disease transmission studies at Colorado State University (CSU) [17–22], with known CWD status, were used for this work. WTD fawns were provided by the Warnell School of Forestry and Natural Resources, University of Georgia, Athens (UGA)—a region in which CWD has not been detected. The fawns were hand-raised and human- and indoor-adapted before being transported directly to the CSU CWD indoor isolation research facility without contact with the native Colorado environment. All deer were housed, handled, monitored daily, anesthetized for sample collections using an intramuscular injection of ketamine (5-8mg/kg) plus metatomidine (0.1–0.1mg/kg) with reversal after sample collection using antisedan (0.175mg/kg), and euthanized via intravenous injection of pentobarital (1ml/4.5kg weight) within 72 hours of stage 2 or 3 disease designation in strict accordance with guidelines for animal care and use provided by the United States Department of Agriculture (USDA), NIH and the Association for Assessment and Accreditation of Laboratory Animal Care International (AAALAC), and CSU International Animal Care and Use Committee (IACUC). All care takers and laboratory personnel were trained to recognize and immediately report behaviors denoted in the CWD clinical scoring system (see below). No animals died prior to euthanasia. All animals were euthanized as per IACUC approved protocols and use of the CWD clinical stage scoring system if developing clinical signs of CWD. Negative controls and animals at stage 0 clinical disease were euthanized to harvest tissues to serve as negative controls in assay assessments (negative controls) or end point collections as per study design (stage 0 clinical disease).

### CWD clinical stage scoring system

All deer were monitored daily for health and clinical presentation of CWD and were assessed for CWD status at study termination. Stage 0: Subclinical; normal behavior and physiological homeostasis. Stage 1: Animal shows a subtle behavioral change. Diurnal rhythms and patterns of sleeping, feeding and activity may be altered. This is only obvious to a caregiver when an individual from a cohort fails to respond to the presence of a caregiver. When aroused, the affected animal may show a decreased level of investigatory behavior and in some cases are hyper-reactive to stimuli. Stage 2: In addition to Stage 1 behavior there is a mild but observable neurological deficit. This is most commonly seen as mild ataxia in the hind-quarters but may include the front legs and head tossing. The animal is fully mobile and continues to interact. Stage 3: Early: In addition to Stage 2 behavior the animal is beginning to show early signs of deterioration (weight loss/altered gait) and continued progression of ataxia. Loss of coat condition becomes more obvious in association with a loss of grooming behavior. Appetite and ability to eat and drink remain intact. Late: Gait abnormalities become pronounced. Locomotion varies from normal to moderately ataxic. There are obvious signs of muscle wasting even though appetite and ability to eat and drink remain intact, and in some cases increase.

### White-tailed deer muscle samples

Muscle tissue analyzed for this study was collected from white-tailed deer (Odocoileus virginianus) between 25–54 months post infection (mpi) that were part of previous CWD studies conducted at Colorado State University (Table 1). All WTD (n = 37) were sourced from the University of George Warnell School of Forestry and Natural Resources and inoculated with various CWD inoculum sources and doses. CWD-positive deer (n = 31) were inoculated either; 1.) orally (PO) with 1g CWD-positive brain tissue (n = 10; designated #7xx; 31.5mpi) [20], 10mg CWD-positive brain tissue (n = 8; designated #10xx; 32mpi) [18], 1 mg CWD-positive brain tissue (n = 2; designated #13xx; 47mpi) [19], 300ng CWD-positive brain tissue (n = 3; designated #13xx; 47mpi) [19], 300ng CWD-positive equivalent saliva (n = 2; designated #13xx; 47mpi) [19]; or 2.) were aerosol-exposed to 2 mL CWD-positive brain tissue (n = 6; designated #8xx; 26mpi) [17]. CWD-negative deer (n = 6) were intravenously-inoculated with CWD negative WTD blood products (n = 1; designated #4xxx; 39mpi) [22], orally-dosed with 126g/130mL feces/urine (n = 2; designated #5xx; 54mpi) [21], aerosol-exposed to 2ml CWD negative white-tailed deer brain (n = 1; designated #8xx; 26mpi) [17], or orally-dosed with 300ng CWD negative white-tailed deer brain/saliva material (n = 2; designated #14xx; 25mpi) [19].

**Table 1:** Detection of prion seeding activity in white-tailed deer hamstring and tongue muscle tissue.

	CWD detection in WTD hamstring and tongue tissues assessed by RT-QuIC (Q) and PMCA/RT-QuIC (PQ)								
						Hamstring		Tongue	
	CWD inoculated WTD								
Animal #	Inoculum	Route	MPI	Genotype	Stage	PQ	Q	PQ	Q
812	5 mg Brain	Aerosol	26	GG	3	-	-	****	**
813	“	“	23	GG	3	***	-	***	**
815	“	“	23	GG	3	***	-	***	***
816	“	“	25	GG	3	**	-	***	***
817	“	“	19	GG	3	***	-	***	**
818	“	“	16.5	GG	3	-	-	***	***
1303	300 ng Brain	PO	22	GG	3	***	**	***	***
1305	1 mg Brain	“	28	GS	0	-	-	***	-
1307	300 ng Brain	“	28	GS	0	-	-	-	-
1309	300 ng Saliva	“	28	GG	2	-	-	***	-
1310	1 mg Brain	“	28	GS	0	-	-	***	-
1313	300 ng Saliva	“	25	GG	3	-	-	***	*
1316	300 ng Brain	“	23	GG	2	-	-	***	-
773	1g Brain	“	30	GS	3	-	-	ND	ND
775	“	“	31	GS	3	***	-	“	“
776	“	“	27	GG	3	***	-	“	“
777	“	“	31.5	GS	2	***	-	“	“
778	“	“	22	GG	3	***	-	“	“
782	“	“	16	GG	3	***	-	“	“
783	“	“	18	GS	0	-	-	“	“
784	“	“	18	GG	3	***	-	“	“
785	“	“	22	GG	3	***	**	“	“
786	“	“	16	GG	1	-	-	“	“
1031	10 mg Brain	“	32	GG	3	-	-	“	“
1076	“	“	22	GG	3	***	-	“	“
1078	“	“	24.5	GG	3	***	-	“	“
1079	“	“	32	GG	3	***	-	“	“
1081	“	“	20	GG	3	-	-	“	“
1082	“	“	24	GG	3	***	-	“	“
1083	“	“	26	GG	0	-	-	“	“
1093	“	“	23	GG	3	***	-	“	“
	**Negative Control WTD**								
814	5 mg Brain	Aerosol	26	GG	0	-	-	“	“
4516	Blood products	IV	21	GG	0	-	-	“	“
502	126g/130 mL Urine/Feces	PO	54	GS	0	-	-	“	“
504	“	“	30	GG	0	-	-	“	“
1437	300 ng Brain/Saliva	“	24	GG	0	ND	ND	-	-
1444	“	“	24	GG	0	“	“	-	-

White-tailed deer hamstring (biceps femoris) and tongue (genioglossus) samples analyzed by RT-QuIC and PMCA with RT-QuIC readout (PQ). Various inoculum sources are indicated, including oral (PO) and aerosol routes. Statistical significance is indicated with asterisks. P values as follows

* <0.05

** < 0.01

*** < 0.001. Clinical stage and genotype for each deer is included.

### Muscle tissue collection/ preparation

Muscle samples measuring approximately 1” x 1” were collected at the time of necropsy and included core sections of the biceps femoris (hamstring), genioglossus (tongue), longissimus dorsi (backstrap), psoas major (tenderloin), gluteus maximus (rump), gastrocnemius (calf), and sternomandibularis (neck). At time of collection, care was taken to avoid harvesting major nerves or lymph nodes with each muscle. Tissues were stored at -80°C until homogenate preparation. Approximately 50-100mg of each tissue was prepared as a 10% (weight/volume) homogenate in PBS in 2ml reinforced homogenate tubes with 1.4mm and 2.8mm zirconium ceramic oxide beads. Samples were homogenized using an Omni Bead Ruptor 24 for 2 minutes and stored at -80°C until testing.

### Serial Protein Misfolding Cyclic Amplification (sPMCA)

sPMCA was performed as previously described [23]. Briefly, normal brain homogenate (NBH) was made from naïve Tg(CerPrP) 5037 mice and used as the substrate for the assay. Ten microliters (10μL) of each 10% muscle tissue homogenate were combined with 90μL NBH for the first round of PMCA. Cycles of sonication (30 seconds) and rest (29 min 30 seconds) were performed for 72 hours in a Misonix sonicator. sPMCA rounds 2–5 consisted of 20μL of the previous round with 50μL NBH with sonication /rest cycle as above for 24 hours. Round 5 PMCA product was diluted to a 10^−3^ final dilution, and 2μL of each sample was mixed with 98μL QuIC Buffer and placed into the RT-QuIC assay in quadruplicate. Each sample was run a minimum of two times for a total of 8 RT-QuIC replicates/muscle sample.

### Real Time Quaking Induced Conversion (RT-QuIC)

RT-QuIC assay was performed as previously described [24]. Briefly, 10% muscle homogenates were diluted to 10^−4^ in 0.1% SDS (sodium dodecyl sulfate, Sigma-Aldrich). Two microliters (2μL) of each sample were added to 98μL RT-QuIC master mix (320 mM NaCl, 1.0 mM EDTA, 10 μM Thioflavin T [ThT, Sigma]) containing truncated Syrian Hamster recombinant protein encoding residues 90–231 (SHrPrP). Each sample was analyzed in quadruplicate on at least two 96 well plates (Greiner Bio-One black, optical-bottom, VWR) for a total of 8 replicates/sample, and placed in a BMG FLUOstar Omega microplate reader for 62 hours at 42°C. Positivity was determined by crossing a threshold of five times the standard deviation from baseline fluorescence and is reported as (1/time to threshold), or amyloid formation rate. A Mann-Whitney unpaired t-test was used against negative controls on each plate to determine statistical significance.

### Immunohistochemistry (IHC)

IHC was performed as previously described [19]. In brief, 5μm sections of formalin fixed muscle tissue mounted on positively charged glass slides were rehydrated in graded alcohols, treated in 88% formic acid, and incubated in citrate buffer with heat-induced epitope retrieval. Slides were placed in 3% hydrogen peroxide to quench endogenous peroxidase activity, then placed in TNB blocking buffer before overnight incubation with BAR-224 (1:750) anti-cervid prion protein mouse monoclonal antibody BAR-224 (1mg/mL; Cayman Chemical) diluted 1:750. Slides were washed and placed in a universal secondary anti-mouse antibody Envision HRP prior to being stained with AEC (3-amino-9-ethylcarbazole; Dako) and counterstained with hematoxylin and bluing reagent.

## Results

Here we assessed prion seeding activity in muscle tissue of CWD-infected white-tailed deer by single (RT-QuIC) and combined use of amplification assays (PMCA with RT-QuIC readout). The focus of our analysis was on muscle tissues commonly prepared for human consumption.

**Fig 1:**
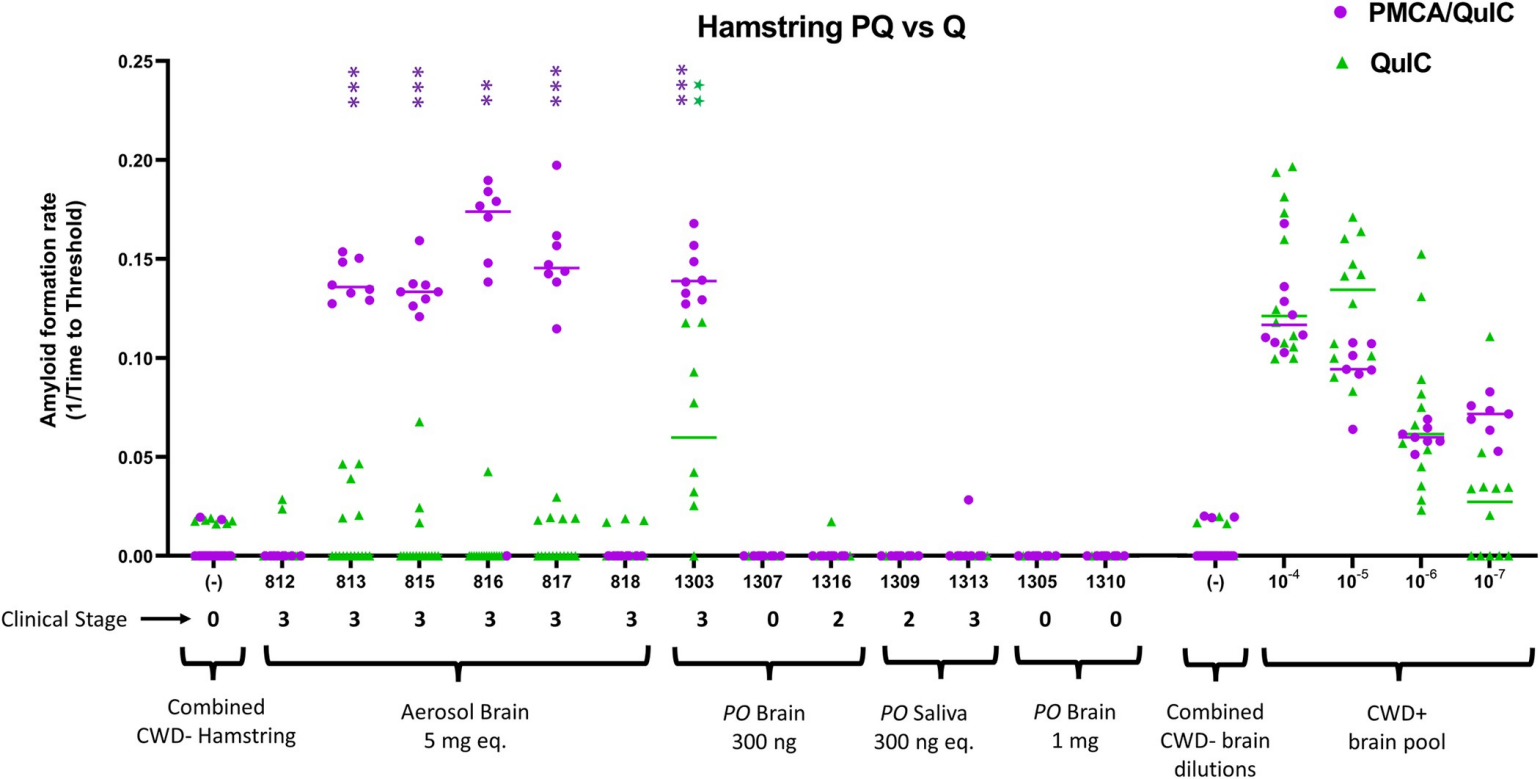
Detection of prion seeding activity in white-tailed deer hamstring (biceps femoris) tissue by single and combine prion amplification assays. Deer designated #8xx (n = 6) were aerosol-exposed to 5mg equivalent CWD-positive brain tissue. Deer designated #13xx (n = 7) were orally-inoculated with 300ng CWD-positive brain tissue (n = 3), 300ng CWD-positive equivalent saliva (n = 2), or 1 mg CWD-positive brain tissue (n = 2). CWD-negative deer (n = 4) were intravenously-inoculated with CWD negative WTD blood products (n = 1; designated #4xxx; 39mpi), orally-dosed with 126g/130mL feces/urine (n = 2; designated #5xx; 54mpi), or aerosol-exposed to 2ml CWD negative white-tailed deer brain (n = 1; designated #8xx; 26mpi). A Mann-Whitney unpaired t-test was used against negative controls on each plate to determine statistical significance. P values as follows: * <0.05, ** < 0.01, *** < 0.001. Clinical stage for each deer is included. Dilutional series of positive cervid brain control is included for rate comparison, as well as a combined dilutional series of negative brain control.

Hamstring muscle was a representative muscle tissue collected at time of necropsy from all white-tailed deer across previous experiments. As we were unable to detect prion PrP^CWD^ deposition in muscle tissues by conventional IHC, hamstring muscle tissue samples (n = 31) were analyzed by RT-QuIC and combined PMCA with RT-QuIC readout (PQ). Of these n = 2/31 (6.45%) were positive for prion seeding activity by traditional RT-QuIC. By employing PMCA with RT-QuIC readout (PQ), prion seeding was detected in n = 17/31 (54.8%) hamstring muscle samples (Fig 1; Table 1). All hamstring muscle tissues from negative or mock-inoculated white-tailed deer (n = 4) remained negative for prion seeding activity (Fig 1; Table 1).

**Fig 2:**
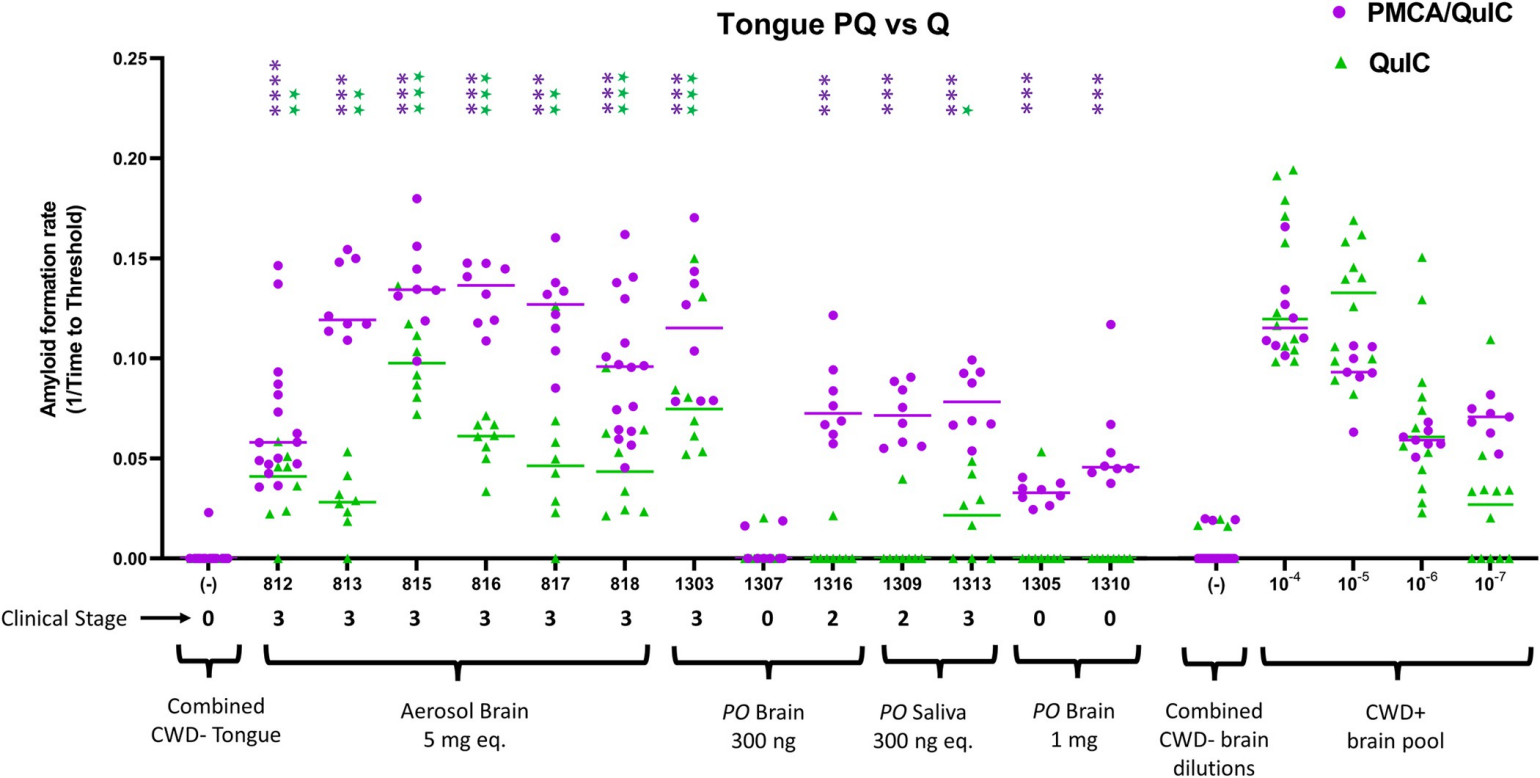
Detection of prion seeding activity in white-tailed deer tongue (genioglossus) tissue by single and combine prion amplification assays. Deer designated #8xx (n = 6) were aerosol-exposed to 5mg equivalent CWD-positive brain tissue. Deer designated #13xx (n = 7) were orally-inoculated with 300ng CWD-positive brain tissue (n = 3), 300ng CWD-positive equivalent saliva (n = 2), or 1mg CWD-positive brain tissue (n = 2). CWD-negative deer (n = 2) were orally-dosed with 300ng CWD negative white-tailed deer brain/saliva material (designated #14xx; 25mpi). A Mann-Whitney unpaired t-test was used against negative controls on each plate to determine statistical significance. P values as follows: * <0.05, ** < 0.01, *** < 0.001, **** < 0.0001. Clinical stage of each deer is included. Dilutional series of positive cervid brain control is included for rate comparison, as well as a combined dilutional series of negative brain control.

Tongue was also routinely collected across all CWD studies at CSU and was analyzed by single and combined amplification assays in this study (Fig 2). By RT-QuIC alone, n = 8/13 (61.5%) tongue samples revealed prion seeding activity. Enhanced seeding detection was demonstrated by incorporation of PQ in 12/13 (92.3%) samples. All tongue tissue from negative or mock-inoculated white-tailed deer (n = 2) remained negative for prion seeding activity (Fig 2; Table 1;).

**Fig 3:**
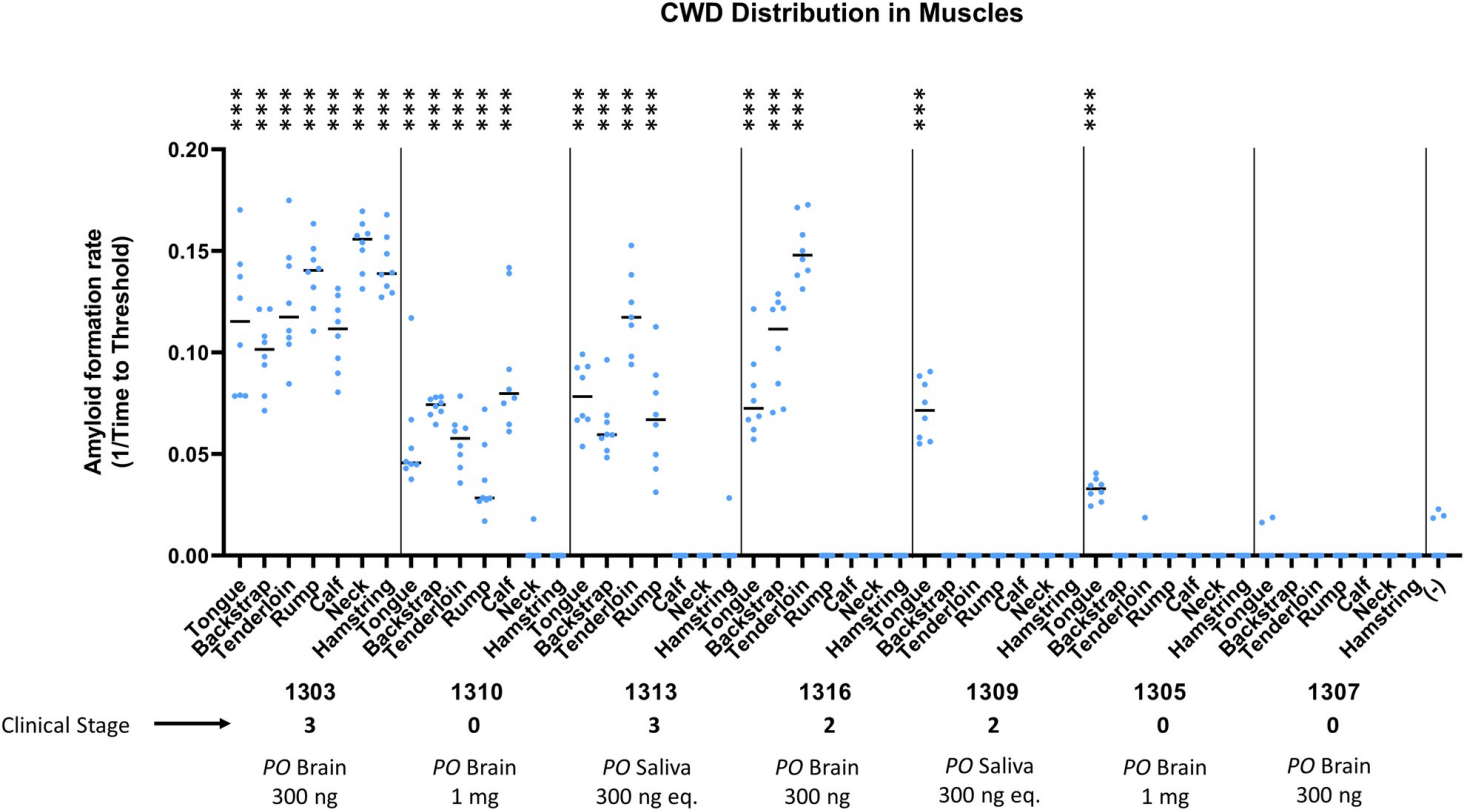
Detection of prion seeding activity in multiple white-tailed deer muscle tissues by combined prion amplification assays. Deer designated #13xx (n = 7) were orally-inoculated with 300ng CWD-positive brain tissue (n = 3), 300ng CWD-positive equivalent saliva (n = 2), or 1mg CWD-positive brain tissue (n = 2). A Mann-Whitney unpaired t-test was used against negative controls on each plate to determine statistical significance. P values as follows: * <0.05, ** < 0.01, *** < 0.001, **** < 0.0001. Clinical stage of each deer is included.

To fill gaps in our knowledge of CWD distribution in muscle tissues throughout the body, five additional muscles were tested by PQ in a subset of 7 deer. The additional muscle samples were collected from CWD orally-inoculated deer (1mg/300ng; designated #13xx) and include backstrap, tenderloin, rump, calf, and neck muscles (Table 2). Of the tissues examined, CWD was detected in tongue n = 6/7 (85.7%), backstrap n = 4/7 (57.1%), tenderloin n = 4/7 (57.1%), rump n = 3/7 (42.9%), calf n = 2/5 (40%), neck n = 1/5 (20%), and hamstring n = 1/7 (14.3%) (Fig 3, Table 2).

**Table 2:** Detection of prion seeding activity in white-tailed deer muscle tissue commonly consumed by humans.

CWD Distribution in WTD muscle tissues assessed by PMCA/RT-QuIC (PQ)								
Deer #		1303	1310	1313	1316	1309	1305	1307
Genotype		GG	GS	GG	GG	GG	GS	GS
**Stage**		3	0	3	2	2	0	0
**Inoculum**		300 ng Brain	1 mg Brain	300 ng Saliva	300 ng Brain	300 ng Saliva	1 mg Brain	300 ng Brain
**Muscle**	Tongue	**+**	**+**	**+**	**+**	**+**	**+**	**-**
	Backstrap	**+**	**+**	**+**	**+**	**-**	**-**	**-**
	Tenderloin	**+**	**+**	**+**	**+**	**-**	**-**	**-**
	Rump	**+**	**+**	**+**	**-**	**-**	**-**	**-**
	Calf	**+**	**+**			**-**	**-**	**-**
	Neck	**+**	**-**			**-**	**-**	**-**
	Hamstring	**+**	**-**	**-**	**-**	**-**	**-**	**-**

Various muscle tissue, focusing on tissues commonly consumed by humans, assessed for prion seeding activity by PMCA with RT-QuIC readout. Deer (n = 7) were orally-inoculated with indicated doses of brain or equivalent saliva. Clinical stage and genotype for each deer is included.

## Discussion

As of June 2024, CWD has been detected in free-ranging and captive cervid populations in 34 U.S. States, 5 Canadian provinces, South Korea, Norway, Sweden, and Finland. As CWD’s geographical spread and prevalence rates continue to expand, so does the likelihood of human exposure and consumption of CWD-infected venison. The debate on CWD’s zoonotic potential is still ongoing. Recent infection studies in humanized mice using brain tissue harvested from CWD-infected white-tailed deer demonstrated the presence of the infectious agent [25], and squirrel monkeys (*Saimiri sciureus)* showed clinical signs of CWD after intracerebral and oral exposure [26]. However, studies in cynomolgus macaques (*Macaca fascicularis)*, a non-human primate species considered phylogenetically closer to humans than squirrel monkeys, resulted in a lack of CWD transmission when assessed by both oral and IC routes of inoculation [27]. As studies on zoonotic potential continue, it remains necessary to focus on the presence of CWD in tissues commonly consumed by humans to better understand the impact of CWD’s expanding distribution and prevalence rates among cervid populations on a potential spillover event. Here, we assessed various muscle tissues harvested from white-tailed deer with known CWD status that are commonly consumed by humans, including hamstring, backstrap, tenderloin, rump, calf, neck, and tongue for prion seeding activity (prions). We found prions present in muscle tissue of deer that received 300ng CWD prions, a dose that represents the minimum infectious dose in white-tailed deer [28], and thus likely represents a biologically relevant infectious dose in captive and free-ranging cervids populations.

Cervids can appear healthy for several years after being infected with CWD. In the U.S. 1 in 36 Americans hunts big game, including cervids [29]. Meat from these hunts is typically consumed by a family or small community of families [30]. Thus, if a CWD-infected cervid is harvested, higher concentrations of CWD prions are likely consumed as the meat product is retained within this circle. Here, we denoted the presence of prions within muscle tissue with the clinical stage of each white-tailed deer at the time of necropsy. For these studies, clinical disease is staged using both behavioral and physiological criteria. At stage 0, no physiological or behavioral signs are seen. By stage 1, deer manifest subtle changes to activity and feeding patterns. Moderate ataxia and more obvious behavioral changes are observable by stage 2; ataxia progresses, and physical deterioration begins in stage 3. It is not until late stage 3/early stage 4 clinical disease that an untrained eye would note significant ataxia, neurological deficits, and muscle wasting. Deer included in these studies were euthanized before reaching stage 4 for humane reasons. We found CWD prions in muscle tissues distributed throughout the body in deer lacking signs of clinical disease (stage 0) as assessed by PMCA-QuIC (PQ) (Table 2). It should therefore be noted that deer through stage 3 clinical disease may appear relatively normal to a hunter or other individual not consistently observing their behavior despite PrP^CWD^ presence in muscle tissues throughout the body.

Immunohistochemistry is considered the gold standard for prion detection, yet we noted that none of the muscle samples tested had detectable IHC PrP^CWD^ deposition. In previous studies using immunohistochemistry, CWD in myocytes of the psoas major was only detectable in cervids with a highly positive obex, indicating advanced disease [13]. Here, CWD was detected in 57.1% of psoas major (tenderloin) samples by PQ, which were in stages 0–3 of clinical disease. This suggests that sufficient prion load is present in muscle tissue during early stages of disease to permit detection by use of combined amplification methods, yet detection by conventional IHC methods requires that the deer be in a more advanced clinical stage of disease.

Previous studies have shown enhanced RT-QuIC detection sensitivity (as high as 80% enhancement) by incorporating sample pretreatments such as NaPTA [16]. To negate challenges in gaining consistent homogenous sample resuspension post-NaPTA treatment, we previously demonstrated that combining amplification assays, including the employment of PMCA followed by RT-QuIC readout (PQ), can result in enhanced detection sensitivity [31]. Using PQ, we were able to improve CWD detection in muscle tissues over use of RT-QuIC alone. We did not employ mouse bioassay of muscle tissue. However, using a previous intracranial transgenic mouse bioassay titration of CWD [32], we were able to gain insight into the relative brain equivalence of prion presence in muscle tissue. As seen in Figs 1 and 2, hamstring and tongue samples amplified by RT-QuIC and PMCA with RT-QuIC readout have similar rates of amplification as the CWD brain dilutional series. The mouse bioassay demonstrated the presence of the infectious CWD agent at dilutions 10^−2^ through 10^−6^, which correlates to roughly 300ug-30ng of infectious CWD prions [31]. Our in vitro amplification results suggest the presence of infectious material within muscle tissue, with the caveat that the dose may not be sufficient to initiate prion disease, specifically within the context of human oral consumption.

Prion detection was variable in muscle tissues throughout the body, with the highest concentrations detected in tongue tissue. This could be due to extensive innervation of the tongue, as previous studies with transmissible mink encephalopathy have found PrP^Sc^ deposition in the nerve fascicles of the tongue [33], more specifically within axons between skeletal muscle fascicles and in the lamina propria [34]. As prion shedding occurs in saliva during clinical disease [24], we also considered saliva contamination as a false positive in the detection of CWD in tongue samples. There was, however, no correlation between prion shedding in saliva and CWD positivity in tongue (Denkers *et*. *al*.; in press) [35]. It is also possible that variation in hematogenous spread contributes to the distribution of muscle infection, as deer have detectable prion burden in blood prior to clinical disease [23].

Prion protein (Prnp) genotype has also been known to significantly affect disease course, with the G96S polymorphism resulting in slower disease progression than deer expressing the wild type G96G polymorphism. This difference can be seen in detection of prion muscle distribution between deer 1316 and 1307 (Table 2). These deer were both inoculated orally with 3 doses of 100ng saliva. 1316 was wild type GG and euthanized at 23 months post inoculation, while 1307 was heterozygous GS and euthanized at 28 months post-inoculation. Both animals were infected with CWD and in a subclinical stage of disease, yet 1316 had prion distribution across three muscle tissues and 1307 was negative throughout, suggesting slower peripheral prion accumulation and disease progression. There is, however, variability in prion distribution in muscle tissue regardless of genotype. For example, 1305 and 1310 (Table 2), both expressing G96S, were orally-inoculated with 1mg brain tissue and euthanized at 28 months post inoculation. Prion seeding was demonstrated only in the tongue of 1305, while prion deposition was dispersed in a majority of tissues collected and analyzed from 1310.

The addition of highly sensitive amplification assays has permitted the detection of very small concentrations of CWD in a variety of biological samples including muscle tissue. It is now clear that regardless of clinical status, genotype, or outward symptoms of the deer, prions may be found throughout the body in tissues consumed by humans. It is thus vital to continue to prioritize CWD education programs and the importance of hunter-harvest sample submissions for continued CWD surveillance, as the ongoing expansion of CWD geographical distribution, strain identity, and herd prevalence rates impacts both cervid and human health.

## Supporting information

S1 Raw data Raw RT-QuIC data can be found in PLOS ONE’s supporting information files. (XLSX)
